# MicroRNA-124-3p suppresses mouse lip mesenchymal cell proliferation through the regulation of genes associated with cleft lip in the mouse

**DOI:** 10.1186/s12864-019-6238-4

**Published:** 2019-11-14

**Authors:** Akiko Suzuki, Hiroki Yoshioka, Dima Summakia, Neha G. Desai, Goo Jun, Peilin Jia, David S. Loose, Kenichi Ogata, Mona V. Gajera, Zhongming Zhao, Junichi Iwata

**Affiliations:** 1 Department of Diagnostic and Biomedical Sciences, School of Dentistry, The University of Texas Health Science Center at Houston, 1941 East Road, BBS 4208, Houston, TX 77054 USA; 2 Center for Craniofacial Research, The University of Texas Health Science Center at Houston, Houston, TX USA; 3 Department of Epidemiology, Human Genetics and Environmental Sciences, School of Public Health, The University of Texas Health Science Center at Houston, Houston, TX USA; 4 MD Anderson Cancer Center UTHealth Graduate School of Biomedical Sciences, Houston, TX USA; 5 Center for Precision Health, School of Biomedical Informatics, The University of Texas Health Science Center at Houston, Houston, TX USA; 6 Department of Integrative Biology and Pharmacology, McGovern Medical School, The University of Texas Health Science Center at Houston, Houston, TX USA

**Keywords:** Cleft lip, Gene mutation, Systematic review, Bioinformatics, Genetic association, Craniofacial development, microRNA

## Abstract

**Background:**

Cleft lip (CL), one of the most common congenital birth defects, shows considerable geographic and ethnic variation, with contribution of both genetic and environmental factors. Mouse genetic studies have identified several CL-associated genes. However, it remains elusive how these CL-associated genes are regulated and involved in CL. Environmental factors may regulate these genes at the post-transcriptional level through the regulation of non-coding microRNAs (miRNAs). In this study, we sought to identify miRNAs associated with CL in mice.

**Results:**

Through a systematic literature review and a Mouse Genome Informatics (MGI) database search, we identified 55 genes that were associated with CL in mice. Subsequent bioinformatic analysis of these genes predicted that a total of 33 miRNAs target multiple CL-associated genes, with 20 CL-associated genes being potentially regulated by multiple miRNAs. To experimentally validate miRNA function in cell proliferation, we conducted cell proliferation/viability assays for the selected five candidate miRNAs (miR-124-3p, let-7a-5p, let-7b-5p, let-7c-5p, and let-7d-5p). Overexpression of miR-124-3p, but not of the others, inhibited cell proliferation through suppression of CL-associated genes in cultured mouse embryonic lip mesenchymal cells (MELM cells) isolated from the developing mouse lip region. By contrast, miR-124-3p knockdown had no effect on MELM cell proliferation. This miRNA-gene regulatory mechanism was mostly conserved in O9–1 cells, an established cranial neural crest cell line. Expression of miR-124-3p was low in the maxillary processes at E10.5, when lip mesenchymal cells proliferate, whereas it was greatly increased at later developmental stages, suggesting that miR-124-3p expression is suppressed during the proliferation phase in normal palate development.

**Conclusions:**

Our findings indicate that upregulated miR-124-3p inhibits cell proliferation in cultured lip cells through suppression of CL-associated genes. These results will have a significant impact, not only on our knowledge about lip morphogenesis, but also on the development of clinical approaches for the diagnosis and prevention of CL.

## Background

Cleft lip (CL) is one of the most common congenital birth defects, with a prevalence of 1/500 to 1/2500 live births worldwide. Approximately 70% of the cases of CL with/without cleft palate (CL/P) are non-syndromic (isolated CL/P), and the remaining 30% are syndromic, displaying many other clinical symptoms and features. The etiology of CL/P is very complex and multifactorial, resulting from the effect of genetic and environmental factors along with geographic, racial, and ethnic influences [1].

Mouse models are well established and have been extensively used to study the mechanisms of CL. Mouse lip formation is similar to that of humans, and the underlying molecular mechanism is well conserved in mice [2]. Mouse lip development begins at embryonic day (E) 10.0 of embryogenesis, when the surface ectoderm thickens bilaterally on the ventrolateral aspect of the frontonasal process to form the nasal placodes. The frontonasal process then expands around the nasal placodes, forming the nasal pits and the horseshoe-shaped medial and lateral nasal processes. The maxillary process then grows rapidly pushing the nasal pits medially, whereas the ventrolateral growth of the medial nasal process converts the round nasal pits into dorsally pointed slits at E10.5. At this stage, the medial nasal process and the maxillary process, with the lateral nasal process wedged in between them, comprise the upper lip, and the fusion of the lateral and medial nasal processes is initiated. By E11.0, the maxillary and medial nasal processes rapidly grow, pushing the lateral nasal process rostrally and fusing between the maxillary and medial nasal processes to form the upper lip [3]. Any failure in the development of the maxillary and nasal processes leads to CL [4].

Previous mouse genetic studies show that mutations in various genes are associated with orofacial cleft, which includes CL, cleft palate, and midfacial/midline cleft [5]. In addition, environmental factors can cause CL [6]. An increasing number of studies suggest that several CL genetic and epigenetic factors could be grouped according to their common functions (e.g. cell proliferation, differentiation) and pathways (e.g. growth factor signaling pathways). However, it remains elusive how CL-associated genes are regulated by epigenetic factors.

MicroRNAs play important role in the post-transcriptional regulation of protein-coding genes, and their altered expression may lead to various developmental defects and diseases [7, 8]. In order to identify the molecular pathways essential for lip formation from the complex etiology of CL, we conducted a systematic review and mouse genome informatics (MGI) database search, followed by bioinformatic analyses, for both CL-associated genes and their related miRNAs. Candidate miRNAs were further tested experimentally in cell proliferation/survival assays and quantitative RT-PCR analyses of target CL-associated genes. This study will help extract molecular pathways and networks associated with CL from currently available data.

## Results

### Study characteristics

**Fig. 1:**
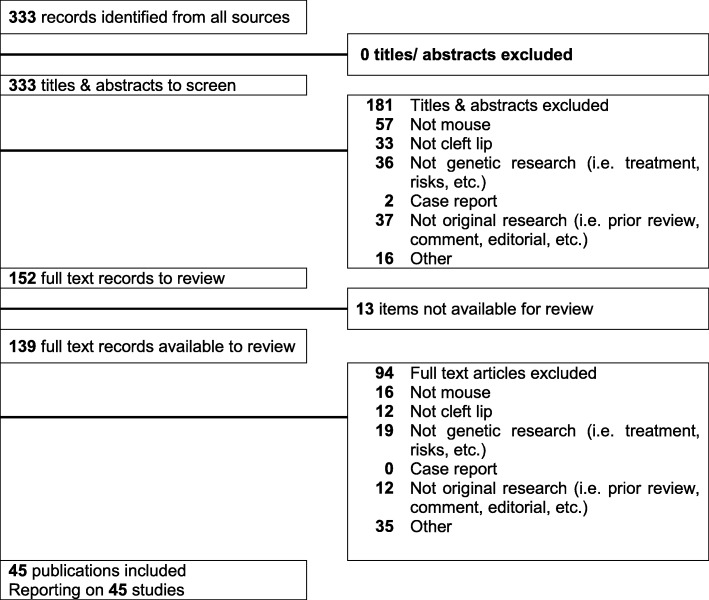
PRISMA flowchart for the selection of studies. A graphical representation of the flow of citations reviewed in the course of the systematic review is provided, using a PRISMA flow diagram

**Fig. 2:**
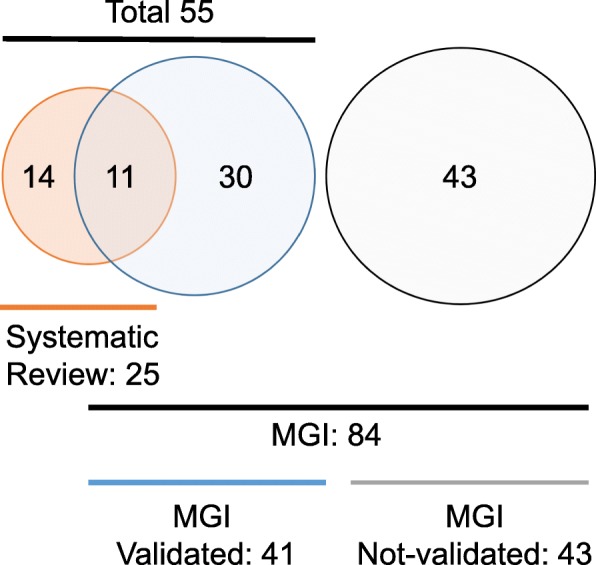
Venn diagram of the mouse cleft lip study

In this study, we focused on CL; therefore, we included cleft lip only (CLO) and cleft lip and palate (CLP), but excluded midline cleft and cleft palate only (CPO). Our extensive literature search resulted in a total of 333 manuscripts. After screening the titles and abstracts of the articles, 152 studies were considered suitable for full-text review to identify the relevant articles; this initial screening was conducted by two screeners independently. As a result, we identified 45 eligible studies that were designed for the collection of causative genes for mouse CL (Fig. 1 and Additional file 1). In these studies, a total of 25 genes [17 single gene mutants and six compound mutants (6 × 2 = 12 genes), with four duplicated genes excluded] and four spontaneous mouse lines with unknown mutation loci were validated as CL genes after the full-text review. In addition, we searched the MGI database, which stores collective information for mouse phenotypes, using the term “cleft lip”; 84 mouse lines were identified in this search. The 43 genes or alleles (51.2%) listed in the MGI database were not validated as CL genes because they were either a reporter gene, a *Cre* expression mouse line, had no CL phenotype, were a duplicate, or were excluded from the CL-associated gene list. As a result, a total of 41 genes [33 genes from single gene mutants and 8 genes from compound mutants after excluding six duplicated genes; 48.8%] were identified as CL-associated genes in the MGI database (Fig. 2).

The bibliographies of highly pertinent articles were further examined to avoid any errors introduced with the systematic review. As a result, we found a total of 55 genes as CL-associated genes. Among them, a total of 39 genes were identified in mice with CL/P resulting from a single gene deficiency (Table 1). There are nine spontaneous CL/P mouse lines (four genes after excluding any duplicated genes; five mouse lines with spontaneous mutations in CL-associated genes and four mouse lines with spontaneous mutations in unknown gene and loci). The penetrance of CL/P in spontaneous mouse lines is quite low (less than 40%) (Table 2). Ten compound mutant mice (mice with two mutant genes; 12 genes after excluding any duplicated genes) exhibited CL (Table 3). Among these 55 CL-associated genes, 20.0% (11 out of 55 genes) were common in the systematic review and MGI database search. There were 14 genes (25.5%, 14 out of 55 genes) and 30 genes (54.5%, 30 out of 55 genes) uniquely identified through the systematic review and MGI search, respectively (Fig. 2).

**Table 1:** Single gene mutant mice with cleft lip

No	Gene symbol	Gene name	Reference	PMID	Note	Cleft type
1	*Bmp4*	bone morphogenic protein 4	[9]	15716346	*Nestin-Cre;Bmp4*^*F/n*^ cKO mice show unilateral CL.	CLO
2	*Bmpr1a*	bone morphogenic protein receptor, type 1A	[9]	15716346	*Nestin-Cre;Bmpr1a* cKO mice show bilateral CL and CP.	CLP
3	*Cdc42*	cell division cycle 42	[10]	24056078	*Wnt1-Cre;Cdc42* cKO mice show either unilateral or bilateral CL at 10% and CP at 100%.	CLP or CPO
4	*Clpex*	cleft lip and palate, exencephaly	[11]	21515572	Homozygous mutant mice show several types of facial clefting (midfacial cleft and bilateral CL) and CP	midfacial cleft and CLP
5	*Cplane1*	ciliogenesis and planar polarity effector 1	[12]	25877302	Homozygous mutant mice show CL and CP.	CLP
6	*Cplane 2 (aka Rsg1)*	ciliogenesis and planar polarity effector 2	[13]	25807483	Homozygous mutants show CL. Mutation is ENU-induced single point mutation.	CLO
7	*Ctnnb1*	catenin, beta1	[14]	22354888	*Pitx1-Cre;Ctnnb1*^*dex3/dex3*^ cKO (gain of function) and *Pitx1-Cre;Ctnnb1*^*dex2–6/dex2–6*^ cKO (loss of function) mice show CL and CP.	CLP
8	*Dzip1l*	DAZ interacting protein 1-like	[15]	28530676	Homozygous null mutant mice show bilateral CL and CP.	CLP
9	*Ednrb*	endothelin receptor type B	[16, 17]	8722795; 17693063	Homozygous null mutant mice show CL at 27% and CP at 83%.	CLP or CPO
10	*Ermp1*	endoplasmic reticulum metallopeptidase 1	[13]	25807483	Homozygous mutant mice show CL and CP. Mutation is ENU-induced single point mutation.	CLP
11	*Esrp1*	epithelial splicing regulatory protein 1	[18]	26371508	Homozygous null mutant mice show CL and CP at 100%.	CLP
12	*Ext1*	exostoses 1	[19]	19509472	*Wnt1-Cre;Ext1* cKO mice show CL and CP.	CLP
13	*Folr1 (aka Folbp1)*	folate receptor 1 (adult)	[20]	12854656	Homozygous null mutant mice show bilateral CL at 43%, unilateral CL at 32%, and CP at 51%. Some embryos show failure of the mandibular process, resulting in mandibular cleft.	CLP or CLO
14	*Gldc*	glycine decarboxylase	[13]	25807483	Homozygous mutant mice show midfacial cleft or CL and CP. Mutation is ENU-induced single point mutation.	midfacial cleft or CLP
15	*Kif7*	kinesin family member 7	[13]	25807483	Homozygous mutant mice show CL or CP. Mutation is ENU-induced single point mutation.	CLO, CLP, or CPO
16	*Lgl*	legless	[21, 22]	3406741; 2313245	*Lgl*^*Tg/Tg*^ (deletion transgenic) mice show midfacial cleft or CL and CP at 40%.	CLP or midfacial cleft and CP
17	*Lrp6*	low density lipoprotein receptor-related protein 6	[3, 23]	19700620; 19653321	Homozygous null mutant mice show either bilateral or unilateral CL and CP at 100%.	CLP
18	*Mirc1 (aka miR-17-92)*	microRNA cluster 1	[24]	24068957	Homozygous null mutant mice show bilateral CL/P at 32.4% and unilateral CL/P at 17.7%. 44% of mutant mice show mandibular cleft.	CLP
19	*Mks1*	Meckel syndrome, type 1	[25, 26]	21045211; 23454480	Homozygous null mutant mice show CL and/or CP.	CLO, CLP, or CPO
20	*Myh10*	myosin, heavy polypeptide 10, non-muscle	[13]	25807483	Homozygous mutant mice show CL. Mutation is ENU-induced single point mutation.	CLO
21	*Nosip*	nitric oxide synthase interacting protein	[27]	25546391	*Nosip* null mice exhibit unilateral CL and CP (48.6%; as a mild phenotype) and midfacial cleft with CP (28.6%; as a severe phenotype).	CLP, midfacial cleft and CP
22	*Pbx1*	pre B cell leukemia homeobox 1	[28]	29797482	*Foxg1-Cre;Pbx1* cKO mice show CPO at 33%, either unilateral or bilateral CL and CP at 62%, and unilateral CLO at 5%.	CLO, CLP, or CPO
23	*Ph*	patch deletion region	[29]	Rasberry and Cattanach, 1994 Mouse Genome, 92 (3):504–505	Homozygous mutant mice show facial cleft or CL.	midfacial cleft, CLO, or CLP
24	*Porcn*	porcupine O-acyltransferase	[30]	25451153	*Wnt1-Cre;Porcn*^*F/Y*^ cKO mice show CL at 100% and CP. *Rx3-Cre;Porcn*^*F/Y*^ cKO mice show bilateral CL and CP. *Wnt1-Cre;Rx3-Cre;Porcn*^*F/Y*^ cKO mice show CL and CP at 100%.	CLO or CLP
25	*Ptch1*	patched 1	[31]	23900075	*Wnt1-Cre;Ptch1* cKO mice show CL or midfacial cleft at E12.5. Embryos die by E12.5.	CL or midfacial cleft
26	*Ptpn11*	protein tyrosine phosphatase, non-receptor type 11	[32]	19706403	*Wnt1-Cre;Ptpn11*^*Tg/+*^ (gain of function) mice show CL and CP at 21%.	CLP
27	*Rpgrip1l*	Rpgrip1-like	[33–35]	17553904; 17558409; 21677750	Homozygous null mutant mice show CL.	CLO
28	*Satb2*	special AT-rich sequence binding protein 2	[36, 37]	16960803; 16751105	Homozygous null mutant mice show CL and CP.	CLP
29	*Sox11*	SRY-box 11	[38, 39]	15254231; 26826126	Homozygous null mutant mice and *EIIa-Cre;Sox11* cKO mice show either unilateral or bilateral CL at 70% and either anterior or complete CP at 100%.	CLP or CPO
30	*Sp8*	trans-acting transcription factor 8	[40]	23872235	*FoxG1-Cre;Sp8 c*KO mice (5 out of 13) exhibit CLO.	CLO
31	*Tbc1d32*	TBC1 domain family, member 32	[13]	25807483	Homozygous mutant mice show CL and CP. Mutation is ENU-induced single point mutation.	CLO or CLP
32	*Tbx1*	T-box 1	[41]	19557177	*Ap2a*^*IRESCre/+*^*;COET* conditional *Tbx1* overexpression mice exhibit bilateral CL. No information about CP. The phenotype was rescued by overexpression of *Smad1* (*Ap2a*^*IRESCre*^*/*^*+*^*;COET;Fsmad1*).	CLO or CLP
33	*Tfap2a*	transcription factor AP-2, alpha	[42]	25381013	*Tfap2a*^*null/neo*^ mice show bilateral CL and CP at 100%.	CLP
34	*Tgfbr1 (aka Alk5)*	transforming growth factor, beta receptor I	[43]	18586087	*Nestin-Cre;Tgfbr1* cKO mice show either unilateral or bilateral CL at 64%. No information about CP.	CLO or CLP
35	*Tmem107*	transmembrane protein 107	[44, 45]	22698544; 28954202	Homozygous mutant mice show CL and CP at 14%.	CLP
36	*Trp53*	transformation related protein 53	[46]	25119037	*CMV-Cre;Trp53*^*LSL-25.26.53.54/+*^ mice show CL and CP.	CLP
37	*Trp63*	transformation related protein 63	[47]	18634775	Homozygous null mutant mice show bilateral CL and CP at 100%.	CLP
38	*Wdr19 (aka Ift144)*	WD repeat domain 19	[48]	22228095	Homozygous mutant mice show bilateral CL and CP. Mutation is ENU-induced single point mutation.	CLP
39	*Wnt9b*	wingless-type MMTV integration site family, member 9B	[49]	21982646	*Foxg1-Cre*^*/+*^*;Wnt9b* cKO mice show bilateral CL at 59% and CP.	CLO or CLP

CLO, cleft lip only; CLP, cleft lip and cleft palate; CPO, cleft palate only

**Table 2:** Spontaneous mutant mice with cleft lip

No	Gene symbol	Gene name	Reference	PMID	Note	Cleft type
1	*Clf2*	cleft lip 2	[50]	7601909	Homozygous mutant mice show CL and CP at higher incidence.	CLP
2	*Rpl38*	ribosomal protein L38	[51, 52]	10889952; 21529712	Heterozygous mutant mice show CL and/or CP.	CLO, CLP, or CPO
3	*Tbx10*	T-box 10	[53, 54]	5297683; 15118109	Homozygous *Tbx10*^*Tg/Tg*^ (gain of function) mice show either unilateral or bilateral CL and CP.	CLP
4	*Wnt9b (aka Clf1)*	wingless-type MMTV integration site family, member 9B	[55]	16998816	Homozygous null mutant mice show either unilateral or bilateral CL with/without CP.	CLO or CLP
5	*Zeb1*	zinc finger E-box binding homeobox 1	[56, 57]	13539273; 10669096	Homozygous mutant mice show either unilateral or bilateral and either complete or incomplete CL and CP. Twirler is mouse line name.	CLP
6	*A/HeJ*	Not gene	[58]	7202260	10% mice show CL/P.	CLO or CLP
7	*A/J*	Not gene	[58, 59]	7202260; 7394720	10% mice show CL/P.	CLO or CLP
8	*A/Wysn*	Not gene	[58]	7202260	20–30% mice show CL/P.	CLO or CLP
9	*CL/Fr*	Not gene	[59–61]	5538410; 7102571; 7394720	20–40% mice show CL/P. The cleft frequency depends on the colony.	CLO or CLP

CLO, cleft lip only; CLP, cleft lip and cleft palate; CPO, cleft palate only

**Table 3:** Compound mutant mice with cleft lip

No	Gene symbol	Gene name	Reference	PMID	Note	Cleft type
1	*Bbs7 & Ift88*	Bardet-Biedl syndrome 7 & intraflagellar transport 88	[62]	22228099	*Bbs7*^*−/−*^*;Ift88*^*orpk*^ double mutant mice exhibit CL at E12.5. No information about cleft palate at later stages. The single mutant mice do not show CL nor CP. *Ift88*^*orpk*^ is a hypomorphic allele.	CLO or CLP
2	*Fgf8* & *Tfap2*	fibroblast growth factor 8 & transcription factor AP-2, alpha	[42]	25381013	*Tfap2*^*null/neo*^*;Fgf8*^*+/−*^ mice show bilateral CL and CP in 10/18 and unilateral CL/P in 8/10. This compound mutant mouse is a rescue model of *Tfap2a*^*null/neo*^ mice.	CLP
3	*Gdf1* & *Nodal*	growth differentiation factor 1 & nodal	[63]	16564040	*Gdf1*^*−/−*^*;Nodal*^*+/−*^ mutant mice show CL at 68% at E13.5.	CLO
4	*Hhat* & *Ptch1*	hedgehog acyltransferase & patched 1	[64]	24590292	*Hhat*^*Tg(Tfap2a-Cre)/+*^*;Ptch1*^*+/−*^ double heterozygous mice show CL and primary palate cleft at E12.5.	CLP
5	*Lrp6* & *Rspo2*	low density lipoprotein receptor-related protein 6 & R-spondin 2	[65]	21237142	*Lrp6*^*+/−*^*;Rspo2*^*−/−*^ mutant mice show CL and CP in 1/6 or CPO in 5/6.	CLP or CPO
6	*Mirc1* & Mirc3 (aka miR-17-92 & miR-106b-25)	microRNA cluster 1 & microRNA cluster 3	[24]	24068957	*Mirc1*^*null/null*^*;Mirc3*^*null/nul*^ mutant mice show bilateral CL and CP in 100%, and mandibular cleft at 100%. *Mirc1*^*null/null*^*;Mirc3*^*null/+*^ mutant mice show bilateral CL/P in 67.5% and unilateral CL/P at 12.5%, and mandibular cleft at 57.5%.	CLP
7	*Msx1* & *Pax9*	msh homeobox1 & paired box 9	[66]	20123092	*Msx1*^*−/−*^*;Pax9*^*−/−*^ double KO mice show either unilateral or bilateral CL at 39%, CP and midfacial hypoplasia at 100%.	CLP or CPO
8	*Pbx1* & *Pbx2*	pre B cell leukemia homeobox 1 & pre B cell leukemia homeobox 2	[49]	21982646	*Foxg1-Cre;Pbx1*^*F/F*^*;Pbx2*^*−/−*^ double cKO mice show bilateral CL. *Foxg1-Cre;Pbx1*^*F/F*^*;Pbx2*^*+/−*^ mice show bilateral CL and CP. *Tcfap2a-Cre;Pbx1*^*F/F*^*;Pbx2*^*+/−*^ mice show CL and/or CP. *Pbx1*^*−/−*^*;Pbx2*^*+/−*^ mutant mice show CL and CP.	CLO, CLP, or CPO
9	*Pbx1* & *Wnt9b*	pre B cell leukemia homeobox 1 & wingless-type MMTV integration site family, member 9B	[49]	21982646	*Foxg1-Cre;Pbx1*^*+/−*^*;Wnt9b*^*F/F*^ mice show bilateral CL at 100% and CP.	CLO or CLP
10	*Pbx1* & *Pbx3*	pre B cell leukemia homeobox 1 & pre B cell leukemia homeobox 3	[49]	21982646	*Pbx1*^*−/−*^*;Pbx3*^*+/−*^ mutant mice show either unilateral or bilateral CL and/or CP. *Tcfap2a-Cre;Pbx1*^*F/F*^*;Pbx3*^*+/−*^ mutants show CL and/or CP. *Foxg1-Cre;Pbx1*^*F/F*^*;Pbx3*^*+/−*^ mutants show CL and/or CP.	CLO, CLP, or CPO

CLO, cleft lip only; CLP, cleft lip and cleft palate; CPO, cleft palate only

### Environmental and epigenetic factors

The prevalence of CL is influenced by genetic background, ethnicity, and gender. In addition, maternal conditions (e.g. age, smoking, alcohol consumption, obesity, micronutrient deficiencies) affect CL prevalence. MicroRNAs (miRNAs), short (~ 22 nucleotides) noncoding RNAs [67] that control gene expression at the post-transcriptional level [68], are known to be altered by maternal conditions and environmental factors. To identify miRNAs that can regulate the expression of CL genes, we carried out a miRNA-target gene enrichment analysis for CL-associated genes. With an adjusted *p*-value < 0.2, we identified 33 miRNAs whose target genes were significantly enriched with the CL genes (Table 4). Among them were miR-124-3p and let-7 family members (let-7a-1-3p, let-7b-3p, let-7c-2-3p, let-7f-1-3p), for which previous miRNA profiling indicated a spatiotemporal-specific expression in the medial nasal and maxillary processes during lip development [70]. These results suggest that miR-124-3p and let-7 family members may play crucial role in lip development. Among the miRNA targets, *Zeb1* was the most frequently targeted gene, by 17 out of 33 miRNAs, followed by *Pbx1*, *Pbx3*, *Ptch1*, and *Sox11*, targeted by 16 miRNAs (Table 5). These results suggest that miRNAs may play a crucial role in the pathology of CL through the regulation of CL-associated genes.

**Table 4:** miRNA enrichment analysis of mouse cleft lip genes (FDR < 0.2)

miRNA	# genes	Gene symbols	*p* value	FDR (BH*)
mmu-miR-200a-3p	10	*Ctnnb1*, *Myh10*, *Zeb1*, *Esrp1*, *Pbx1*, *Ptch1*, *Satb2*, *Sox11*, *Tfap2a*, *Tgfbr1*	3.00E-05	0.053
mmu-miR-141-3p	9	*Myh10*, *Zeb1*, *Esrp1*, *Pbx1*, *Ptch1*, *Satb2*, *Sox11*, *Tfap2a*, *Tgfbr1*	1.74E-04	0.062
mmu-miR-196a-5p	6	*Ednrb*, *Pbx1*, *Pbx3*, *Rpgrip1l*, *Rspo2*, *Sox11*	1.41E-04	0.062
mmu-miR-196b-5p	6	*Ednrb*, *Pbx1*, *Pbx3*, *Rpgrip1l*, *Rspo2*, *Sox11*	1.41E-04	0.062
mmu-miR-710	6	*Cdc42*, *Ctnnb1*, *Pbx3*, *Rpgrip1l*, *Satb2*, *Sp8*	1.29E-04	0.062
mmu-miR-101a-3p	10	*Cdc42*, *Msx1*, *Pax9*, *Pbx3*, *Ptch1*, *Rspo2*, *Sox11*, *Tbx1*, *Tgfbr1*, *Zeb1*	4.77E-04	0.072
mmu-miR-101b-3p	9	*Cdc42*, *Msx1*, *Pbx3*, *Ptch1*, *Rspo2*, *Sox11*, *Tbx1*, *Tgfbr1*, *Zeb1*	5.31E-04	0.072
mmu-miR-144-3p	9	*Msx1*, *Pbx3*, *Ptch1*, *Rpgrip1l*, *Rspo2*, *Sox11*, *Tbx1*, *Tgfbr1*, *Zeb1*	2.72E-04	0.072
mmu-let-7a-1-3p	5	*Bmpr1a*, *Cdc42*, *Ctnnb1*, *Lrp6*, *Ptch1*	5.25E-04	0.072
mmu-let-7b-3p	5	*Bmpr1a*, *Cdc42*, *Ctnnb1*, *Lrp6*, *Ptch1*	5.25E-04	0.072
mmu-let-7c-2-3p	5	*Bmpr1a*, *Cdc42*, *Ctnnb1*, *Lrp6*, *Ptch1*	5.25E-04	0.072
mmu-let-7f-1-3p	5	*Bmpr1a*, *Cdc42*, *Ctnnb1*, *Lrp6*, *Ptch1*	5.25E-04	0.072
mmu-miR-98-3p	5	*Bmpr1a*, *Cdc42*, *Ctnnb1*, *Lrp6*, *Ptch1*	5.25E-04	0.072
mmu-miR-181a-5p	11	*Ednrb*, *Ermp1*, *Ptch1*, *Ptpn11*, *Myh10*, *Pax9*, *Pbx1*, *Pbx3*, *Rspo2*, *Sox11*, *Tgfbr1*	7.27E-04	0.081
mmu-miR-466 l	13	*Bmp4*, *Dzip1l*, *Lrp6*, *Pax9*, *Pbx1*, *Pbx3*, *Ptpn11*, *Rspo2*, *Satb2*, *Sox11*, *Tbx1*, *Wnt9b*, *Zeb1*	1.26E-03	0.118
mmu-miR-686	5	*Pbx1*, *Rpgrip1l*, *Tgfbr1*, *Zeb1*, *Ptch1*	1.40E-03	0.124
mmu-miR-320-3p	7	*Bmpr1a*, *Ctnnb1*, *Lrp6*, *Pbx1*, *Pbx3*, *Satb2*, *Tgfbr1*	1.49E-03	0.126
mmu-miR-205-5p	6	*Ext1*, *Lrp6*, *Pax9*, *Pbx1*, *Satb2*, *Zeb1*	1.62E-03	0.131
mmu-miR-491	14	*Cdc42*, *Ermp1*, *Esrp1*, *Fgf8*, *Kif7*, *Mks1*, *Myh10*, *Pax9*, *Pbx2*, *Tbx10*, *Wdr19*, *Wnt9b*, *Zeb1*, *Sox11*	1.76E-03	0.136
mmu-miR-142a-3p	7	*Bmpr1a*, *Ctnnb1*, *Myh10*, *Sox11*, *Sp8*, *Tgfbr1*, *Zeb1*	2.45E-03	0.139
mmu-miR-302c	5	*Ednrb*, *Pbx3*, *Tgfbr1*, *Wnt9b*, *Zeb1*	2.39E-03	0.139
mmu-miR-669b	5	*Cdc42*, *Ext1*, *Pbx2*, *Rpgrip1l*, *Sox11*	2.39E-03	0.139
mmu-miR-669f	9	*Cdc42*, *Gldc*, *Msx1*, *Pax9*, *Pbx1*, *Pbx3*, *Rspo2*, *Satb2*, *Tgfbr1*	2.05E-03	0.139
mmu-miR-124	16	*Bmpr1a*, *Ctnnb1*, *Ednrb*, *Esrp1*, *Folr1*, *Gldc*, *Hhat*, *Ift88*, *Lrp6*, *Myh10*, *Pax9*, *Pbx1*, *Ptpn11*, *Rspo2*, *Zeb1*, *Tgfbr1*	2.91E-03	0.149
mmu-miR-124-3p	13	*Cdc42*, *Pbx3*, *Sp8*, *Bmpr1a*, *Ednrb*, *Ermp1*, *Esrp1*, *Ift88*, *Lrp6*, *Myh10*, *Ptpn11*, *Tgfbr1*, *Zeb1*	2.95E-03	0.149
mmu-miR-374c-5p	7	*Esrp1*, *Myh10*, *Pbx3*, *Ptch1*, *Ptpn11*, *Sp8*, *Zeb1*	3.34E-03	0.165
mmu-miR-425-5p	6	*Pbx1*, *Ptch1*, *Ptpn11*, *Rpgrip1l*, *Tgfbr1*, *Satb2*	3.79E-03	0.174
mmu-miR-673-5p	5	*Sox11*, *Ctnnb1*, *Pax9*, *Rpgrip1l*, *Sp8*	3.81E-03	0.174
mmu-miR-142-5p	6	*Cdc42*, *Ctnnb1*, *Pax9*, *Pbx3*, *Rpgrip1l*, *Trp63*	3.64E-03	0.174
mmu-miR-543-3p	6	*Ermp1*, *Myh10*, *Pbx1*, *Pbx3*, *Ptch1*, *Rspo2*	4.58E-03	0.194
mmu-miR-340-5p	24	*Bbs7*, *Bmp4*, *Bmpr1a*, *Cdc42*, *Ermp1*, *Esrp1*, *Gldc*, *Lrp6*, *Mks1*, *Msx1*, *Pbx1*, *Pbx2*, *Pbx3*, *Rpgrip1l*, *Rspo2*, *Sox11*, *Tgfbr1*, *Tmem107*, *Trp53*, *Trp63*, *Wdr19*, *Zeb1*, *Myh10*, *Ptch1*	4.98E-03	0.198
mmu-miR-23a-3p	8	*Ednrb*, *Esrp1*, *Pax9*, *Pbx1*, *Rpgrip1l*, *Satb2*, *Sox11*, *Zeb1*	5.13E-03	0.198
mmu-miR-23b-3p	8	*Ednrb*, *Esrp1*, *Pax9*, *Pbx1*, *Rpgrip1l*, *Satb2*, *Sox11*, *Zeb1*	5.07E-03	0.198

* FDR (false discovery rate): the *p-*values were corrected using the Benjamini–Hochberg multiple test correction [69]

**Table 5:** Mouse cleft lip genes targeted by multiple miRNAs (≥ 2) in the miRNA enrichment analysis (FDR < 0.2)

Gene	# miRNA	miRNAs
*Zeb1*	17	miR-124, miR-340-5p, miR-491, miR-101a-3p, miR-101b-3p, miR-124-3p, miR-141-3p, miR-142a-3p, miR-144-3p, miR-200a-3p, miR-205-5p, miR-23a-3p, miR-23b-3p, miR-374c-5p, miR-686, miR-302c, miR-466 l
*Pbx1*	16	miR-124, miR-340-5p, miR-141-3p, miR-181a-5p, miR-196a-5p, miR-196b-5p, miR-200a-3p, miR-205-5p, miR-23a-3p, miR-23b-3p, miR-320-3p, miR-425-5p, miR-543-3p, miR-686, miR-466 l, miR-669f
*Pbx3*	16	miR-340-5p, miR-101a-3p, miR-101b-3p, miR-124-3p, miR-144-3p, miR-181a-5p, miR-196a-5p, miR-196b-5p, miR-320-3p, miR-374c-5p, miR-543-3p, miR-710, miR-142-5p, miR-302c, miR-466 l, miR-669f
*Ptch1*	16	miR-340-5p, miR-101a-3p, miR-101b-3p, miR-141-3p, miR-144-3p, miR-181a-5p, miR-200a-3p, miR-374c-5p, miR-425-5p, miR-543-3p, miR-686, let-7a-1-3p, let-7b-3p, let-7c-2-3p, let-7f-1-3p, miR-98-3p
*Sox11*	16	miR-340-5p, miR-491, miR-101a-3p, miR-101b-3p, miR-141-3p, miR-142a-3p, miR-144-3p, miR-181a-5p, miR-196a-5p, miR-196b-5p, miR-200a-3p, miR-23a-3p, miR-23b-3p, miR-673-5p, miR-466 l, miR-669b
*Tgfbr1*	15	miR-124, miR-340-5p, miR-101a-3p, miR-101b-3p, miR-124-3p, miR-141-3p, miR-142a-3p, miR-144-3p, miR-181a-5p, miR-200a-3p, miR-320-3p, miR-425-5p, miR-686, miR-302c, miR-669f
*Cdc42*	14	miR-340-5p, miR-491, miR-101a-3p, miR-101b-3p, miR-124-3p, miR-710, miR-142-5p, miR-669b, miR-669f, let-7a-1-3p, let-7b-3p, let-7c-2-3p, let-7f-1-3p, miR-98-3p
*Ctnnb1*	12	miR-124, miR-142a-3p, miR-200a-3p, miR-320-3p, miR-673-5p, miR-710, miR-142-5p, let-7a-1-3p, let-7b-3p, let-7c-2-3p, let-7f-1-3p, miR-98-3p
*Rpgrip1l*	12	miR-340-5p, miR-144-3p, miR-196a-5p, miR-196b-5p, miR-23a-3p, miR-23b-3p, miR-425-5p, miR-673-5p, miR-686, miR-710, miR-142-5p, miR-669b
*Lrp6*	11	miR-124, miR-340-5p, miR-124-3p, miR-205-5p, miR-320-3p, miR-466 l, let-7a-1-3p, let-7b-3p, let-7c-2-3p, let-7f-1-3p, miR-98-3p
*Pax9*	11	miR-124, miR-491, miR-101a-3p, miR-181a-5p, miR-205-5p, miR-23a-3p, miR-23b-3p, miR-673-5p, miR-142-5p, miR-466 l, miR-669f
*Rspo2*	11	miR-124, miR-340-5p, miR-101a-3p, miR-101b-3p, miR-144-3p, miR-181a-5p, miR-196a-5p, miR-196b-5p, miR-543-3p, miR-466 l, miR-669f
*Bmpr1a*	10	miR-124, miR-340-5p, miR-124-3p, miR-142a-3p, miR-320-3p, let-7a-1-3p, let-7b-3p, let-7c-2-3p, let-7f-1-3p, miR-98-3p
*Myh10*	10	miR-124, miR-340-5p, miR-491, miR-124-3p, miR-141-3p, miR-142a-3p, miR-181a-5p, miR-200a-3p, miR-374c-5p, miR-543-3p
*Satb2*	10	miR-141-3p, miR-200a-3p, miR-205-5p, miR-23a-3p, miR-23b-3p, miR-320-3p, miR-425-5p, miR-710, miR-466 l, miR-669f
*Esrp1*	9	miR-124, miR-340-5p, miR-491, miR-124-3p, miR-141-3p, miR-200a-3p, miR-23a-3p, miR-23b-3p, miR-374c-5p
*Ednrb*	8	miR-124, miR-124-3p, miR-181a-5p, miR-196a-5p, miR-196b-5p, miR-23a-3p, miR-23b-3p, miR-302c
*Ptpn11*	6	miR-124, miR-124-3p, miR-181a-5p, miR-374c-5p, miR-425-5p, miR-466 l
*Ermp1*	5	miR-340-5p, miR-491, miR-124-3p, miR-181a-5p, miR-543-3p
*Msx1*	5	miR-340-5p, miR-101a-3p, miR-101b-3p, miR-144-3p, miR-669f
*Sp8*	5	miR-124-3p, miR-142a-3p, miR-374c-5p, miR-673-5p, miR-710
*Tbx1*	4	miR-101a-3p, miR-101b-3p, miR-144-3p, miR-466 l
*Gldc*	3	miR-124, miR-340-5p, miR-669f
*Pbx2*	3	miR-340-5p, miR-491, miR-669b
*Wnt9b*	3	miR-491, miR-302c, miR-466 l
*Bmp4*	2	miR-340-5p, miR-466 l
*Ext1*	2	miR-205-5p, miR-669b
*Ift88*	2	miR-124, miR-124-3p
*Mks1*	2	miR-340-5p, miR-491
*Tfap2a*	2	miR-141-3p, miR-200a-3p
*Trp63*	2	miR-340-5p, miR-142-5p
*Wdr19*	2	miR-340-5p, miR-491

### Experimental validation

**Fig. 3:**
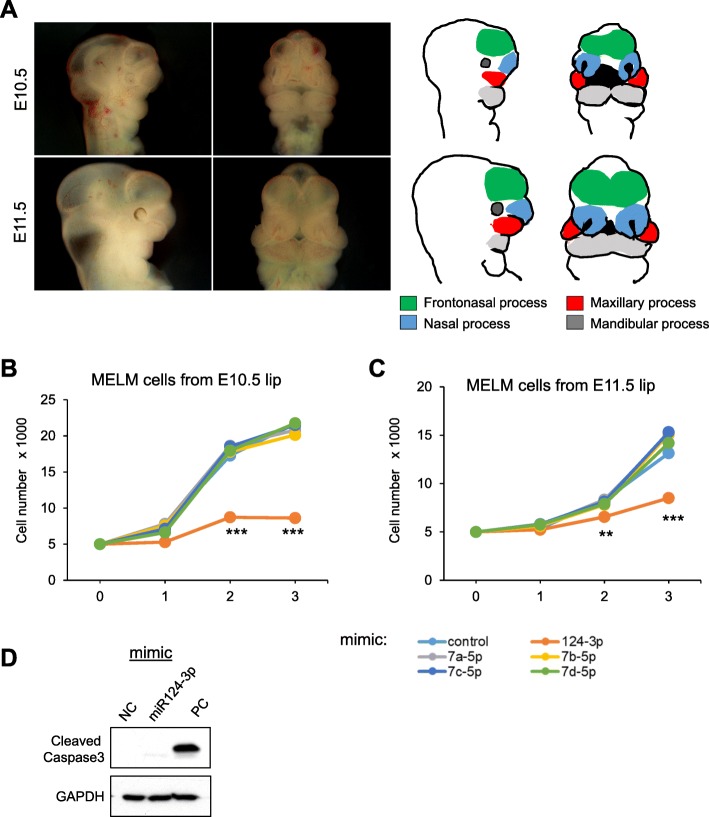
Effect of overexpression of the predicted miRNAs on cell proliferation. **a** Side (left) and frontal (right) view of mouse embryos at E10.5 and E11.5. The drawings on the right show a mouse head at each developmental stage. Color code: frontonasal process, green; maxillary process, red; nasal process, light blue; and mandibular process, gray. **b**, **c** Cell proliferation assays using MELM cells from E10.5 (B) and E11.5 (**c**) lips treated with the indicated miRNAs. Negative control (control, light blue), miR-124-3p (orange), let-7a-5p (gray), let-7b-5p (yellow), let-7c-5p (blue), and let-7d-5p (light green). ** *p* < 0.01, *** *p* < 0.001. **s** Immunoblotting analysis for cleaved caspase 3 in MELM cells treated with negative control (NC), miR-124-3p mimic, and positive control (PC). GAPDH was used as an internal control

**Fig. 4:**
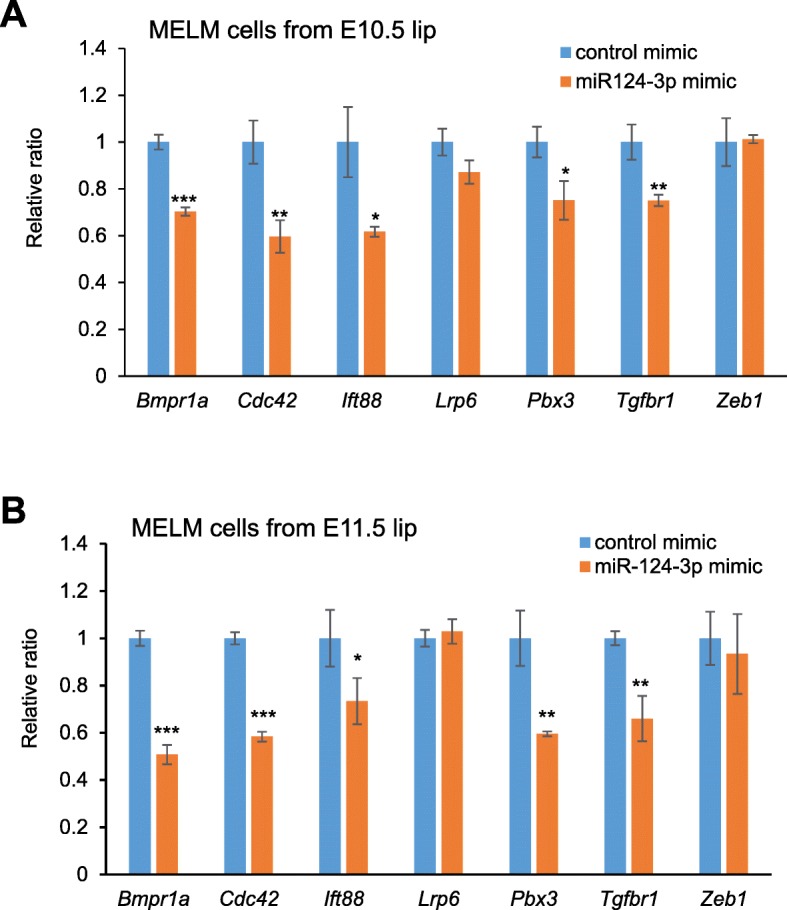
Cleft lip-associated genes suppressed by overexpression of miR-124-3p in MELM cells. **a**, **b** Quantitative RT-PCR for the indicated genes after treatment with negative control (light blue) or miR-124-3p mimic (orange) in MELM cells isolated from E10.5 (**a**) and E11.5 (**b**) developing lip regions. * *p* < 0.05, ** *p* < 0.01, *** *p* < 0.001

miRNAs suppress multiple target mRNAs [71]. Because loss of function of CL-associated genes causes CL in mice, we tested whether overexpression of these miRNAs inhibited cell proliferation through the suppression of target genes. To test this hypothesis, we used primary mouse embryonic upper lip mesenchymal (MELM) cells isolated from the developing upper lip region (Fig. 3a), which were then treated with each miRNA mimic. The miR-124-3p mimic significantly inhibited cell proliferation in MELM cells isolated from the developing lip regions; by contrast, treatment with mimics for let-7a-5p, let-7b-5p, let-7c-5p, and let-7d-5p resulted in no proliferation defect (Fig. 3b, c). We also confirmed that the miR-124-3p mimic did not induce apoptosis (Fig. 3d). To identify target genes regulated by miR-124-3p, we performed quantitative RT-PCR analyses for the predicted target genes in MELM cells after treatment with the miR-124-3p mimic and observed that expression of *Bmpr1a*, *Cdc42*, *Ift88*, *Pbx3* and *Tgfbr1* was significantly downregulated (Fig. 4).

**Fig. 5:**
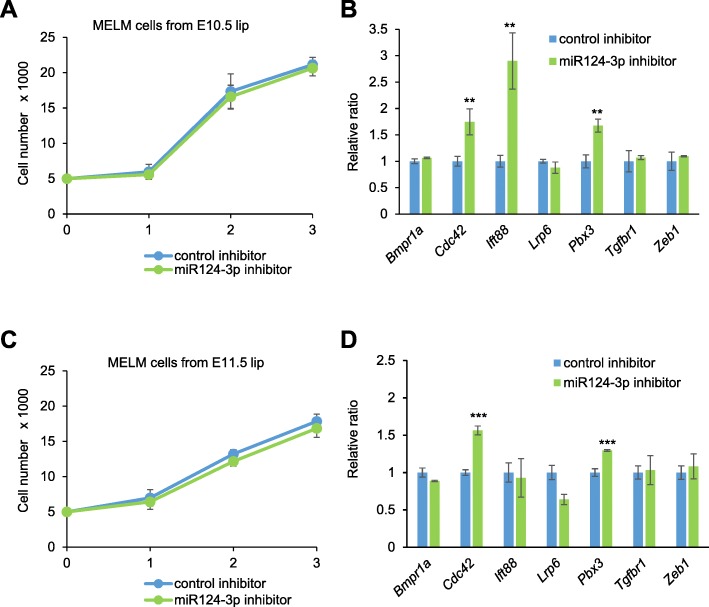
Effect of suppression of miR-124-3p on cell proliferation. **a**, **c** Cell proliferation assays using MELM cells from E10.5 (**a**) and E11.5 (**c**) upper lips treated with negative control (control, light blue) and miR-124-3p inhibitor (green). **b**, **d** Quantitative RT-PCR for the indicated genes after treatment with negative control (light blue) or miR-124-3p inhibitor (green) in MELM cells isolated from E10.5 (**b**) and E11.5 (**d**) developing lip regions. ** *p* < 0.01, *** *p* < 0.001

Next, to examine the effect of loss-of-function of miR-124-3p in cell proliferation and CL-associated gene regulation, we performed cell proliferation assays and quantitative RT-PCR analyses for CL-associated genes in cells treated with a miR-124-3p inhibitor. We found that miR-124-3p inhibition did not affect cell proliferation in MELM cells isolated from either E10.5 or E11.5 maxillary processes (Fig. 5a, c). This indicates that loss-of-function of miR-124-3p has less impact on cell proliferation during lip development. *Cdc42* and *Pbx3*, which were suppressed by miR-124-3p overexpression, were upregulated upon treatment with miR-124-3p inhibitor in MELM cells (Fig. 5b, d), suggesting that the expression of these genes is regulated by miR-124-3p in a dose-dependent manner and that they may be accurate target genes of miR-124-3p in lip development.

**Fig. 6:**
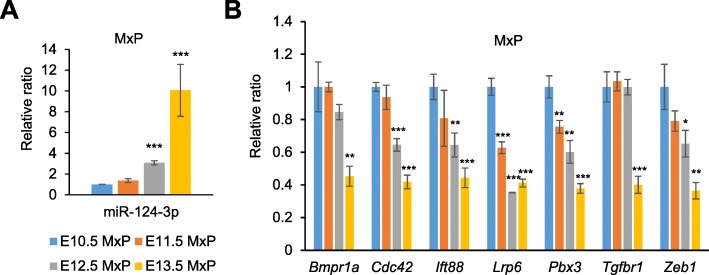
Temporal expression of miR-124-3p and its target genes during lip development. **a**, **b** Expression of miR-124-3p (**a**) and its target genes (**b**) in the maxillary process (MxP) from E10.5 to E13.5. * *p* < 0.05, ** *p* < 0.01, *** *p* < 0.001

Next, we examined when and where miR-124-3p was expressed during normal lip development. Expression of miR-124-3p was slightly upregulated at E12.5, and greatly increased at E13.5, in the maxillary process during lip development (Fig. 6a). The expression of the predicted target genes was anti-correlated with miR-124-3p expression in the maxillary process at E10.5 to E13.5 (Fig. 6b).

**Fig. 7:**
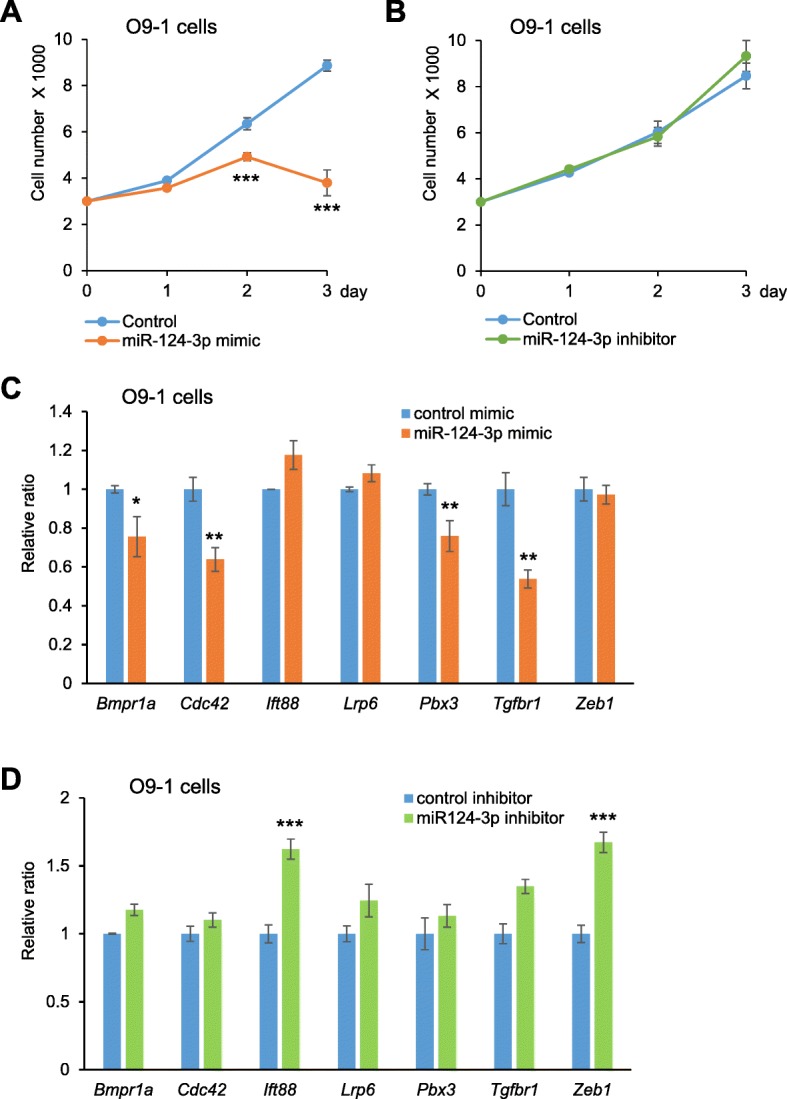
Effect of miR-124-3p in O9–1 cells. **a** Cell proliferation assays in O9–1 cells treated with negative control (light blue) and miR-124-3p mimic (orange). *** *p* < 0.001. **b** Cell proliferation assays in O9–1 cells treated with negative control (light blue) and miR-124-3p inhibitor (green). **c** Quantitative RT-PCR for the indicated genes after treatment with negative control (light blue) or miR-124-3p mimic (orange) in O9–1 cells. * *p* < 0.05, ** *p* < 0.01. **d** Quantitative RT-PCR for the indicated genes after treatment with negative control (light blue) or miR-124-3p inhibitor (green) in O9–1 cells. *** *p* < 0.001

To examine the conservation of these phenotypes in other cell types that are similar to mouse lip cells, we analyzed O9–1 cells, an established cranial neural crest cell line isolated from E8.5 mouse embryos, after treatment with a miR-124-3p mimic. As expected, miR-124-3p strongly suppressed cell proliferation (Fig. 7a). By contrast, the miR-124-3p inhibitor did not alter O9–1 cell proliferation (Fig. 7b), as seen for MELM cells. Next, the expression of the predicted target genes was examined in O9–1 cells in order to compare it with that of MELM cells. We found that expression of *Bmpr1a*, *Cdc42*, *Pbx3*, and *Tgfbr1* was suppressed by the miR-124-3p mimic, as seen in MELM cells (Fig. 6, c, d). In addition, during nasal process development, miR-124-3p overexpression inhibited cell proliferation in primary cells isolated from E11.5 medial nasal processes, as seen for MELM cells. Furthermore, the expression of miR-124-3p and its target genes was similarly changed during nasal process development (Additional file 2).

Taken together, our results indicate that upregulated miR-124-3p results in suppressed cell proliferation through CL-associated gene expression in cultured MELM and O9–1 cells.

## Discussion

CL with or without cleft palate is part of the clinical features of approximately 400 known human syndromes [5]. A significant number of genetic mutations have been reported in CL mouse models. To focus on the CL phenotype, we excluded genes related to cleft palate only and to midline cleft and identified 55 CL genes in mice through a literature review and MGI search.

Recently, a growing number of miRNA profiling studies clarified the contribution of miRNAs to nonsyndromic CL/P [72–74]. The contribution of miRNAs to CL has been elucidated using mice with a deletion of *Dicer*, a crucial enzyme for miRNA maturation [75]. Mice with the *Dicer* deletion in cranial neural crest (CNC) cells and lip mesenchymal cells exhibit severe craniofacial anomalies, including CL, through decreased cell proliferation and increased cell death [76, 77], indicating that mesenchymal miRNAs play essential roles in lip development. By contrast, mice with the *Dicer* deletion in the lip epithelium (*Dicer*^*F/F*^*;K14-Cre* or *Dicer*^*F/F*^*;Shh-Cre* mice: *K14-Cre* and *Shh-Cre* are specifically expressed in the differentiating epithelium) exhibit no CL or craniofacial deformities [78, 79]. This suggests that miRNAs may be less important in the lip epithelium compared to the mesenchyme. However, recent studies indicate that a Dicer-independent pathway exists in the miRNA maturation process [80]. Because the contribution of Dicer-independent miRNAs to lip fusion remains unknown, future genetic studies will identify the role of Dicer-independent miRNAs during lip formation.

In our experimental validation, we validated that miR-124-3p suppresses cell proliferation in cultured mouse lip mesenchymal cells. In nasopharyngeal carcinoma cells, miR-124-3p inhibits cell growth and metastasis formation by targeting STAT3 [81]. By contrast, let-7a-d failed to suppress cell proliferation in cultured lip mesenchymal cells, while let-7a inhibits cell proliferation in gastric cancer cells [82]. Although other miRNAs would potentially regulate the expression of these genes, our miRNA predictions did not reach significance for any other miRNAs. In cases when we did not see a consistent and dose-dependent change with miR-124-3p, these genes’ expression might undergo a more complex regulation by other miRNAs, a combination of miR-124-3p and other miRNAs, or they may be suppressed at the protein translation level. Our results also suggest that each miRNA functions in a cell-specific manner.

There are limited numbers of genetically engineered mice to evaluate the role of individual miRNA in vivo. Currently, miR-17-92 cluster mutant mice exhibit bilateral or unilateral CL through the regulation of the T-box factor genes and fibroblast growth factor (FGF) signaling [24]. In future studies, we will test the role of each miRNA in genetically engineered mice for each candidate miRNA. Moreover, as seen in compound mutant mice with combined gene mutations, an altered miRNA expression profile may contribute to the etiology of CL. For example, the reduction of miR-106b-25 on the miR-17-92 null background results in a more severe cleft phenotype with complete penetrance, indicating that there is a genetic interaction between these two miRNA clusters [24]. Currently, the contribution and distribution of each miRNA, and the interactions between miRNAs, are still largely unknown in lip formation. Our bioinformatic analysis in combination with a systematic literature review and MGI database search is one of the ways to predict functional miRNAs in lip development. In addition, our experimental validation indicates that gain-of-function of miR-124-3p, but not loss-of-function, suppresses cell proliferation through suppression of CL-associated genes in MELM and O9–1 cells. These results are well supported by the fact that mice with loss-of-function mutations in these CL-associated genes exhibit CL.

As there is a discrepancy in the number of studies identified through the systematic review and the MGI search, the systematic review presents some limitations, which may derive from the following: 1) some genes are reported in syndromes that display CL, but CL is not specifically mentioned in the title and abstract; and 2) different terms were used to describe the CL phenotype (e.g. craniofacial anomalies, midfacial deformities). Nonetheless, the advantage of a systematic review is that enables the identification of articles related to topics in a non-biased way. In addition, the current databases fail to provide an accurate list of mouse genes related to the topics searched. For this reason, we conducted both the systematic review and the MGI search in this study and focused on the generation of a list of genes related to CL in mice. This gene list will be useful for future genetic studies as a reference and in the identification of pathways and networks associated with CL.

## Conclusions

The results from this study are important to understand the mechanisms and etiology of CL, to further validate CL-associated genes and their regulation in CL, and to design future clinical applications to prevent and diagnose CL in humans. It has been known that expression of miRNAs is altered by extracellular conditions. Our results suggest that upregulated miR-124-3p may cause CL through the suppression of CL-associated genes. This new knowledge has potential relevance for the pathways and networks of CL-associated genes and miRNAs in the regulation of the development of the lip.

## Methods

### Information sources for the gene search

We followed a guideline set forth by PRISMA (Preferred Reporting Items for Systematic Reviews and Meta-Analyses) [83] for the systematic CL gene search. Public online databases Medline (Ovid), Embase (Ovid), and PubMed (NLM) were searched for articles and information on mouse CL-associated genes. In order to recover any missing data related to CL, we searched Scopus (Elsevier) and the MGI database. RefWorks was used for sorting the references and excluding duplicates from the systematic review, as described previously [84].

### Eligibility criteria for the systematic review

The following inclusion criteria were applied in the selection of the articles:

1. genetic studies for mouse CL;
2. original articles (no review articles, editorials, or comments);
3. published in English;
4. articles specifying the genes responsible for CL in mice.

After the step above, we manually excluded those studies meeting one or more of the following criteria:

1. conducted primarily in other species;
2. describing environmental factors for CL instead of genetic factors.

### Search strategy to identify the studies

A systematic literature search was conducted independently by two screeners using the Medline (Ovid), PubMed (NLM), and Embase (Ovid) databases. To conduct the search, Medical Subject Headings (MeSH) terms were developed, as described previously [85]. Different combinations and variations of the term ‘CL’ (i.e. CL, CL/P, CL and palate) were searched along with other terms such as ‘mice’ (or ‘mouse’), ‘genetics’, and ‘mutation’. Additionally, the bibliographies of the relevant articles were manually examined in Scopus (Elsevier) to retrieve studies that were not identified in the database searches.

### Study design and case selection

RefWorks (ProQuest) and systematic review Excel workbooks were used to store and track all citations found in the search process and to eliminate duplicates. The Kappa statistic was used to determine the level of agreement between the two screeners. Full-text articles for which there was a disagreement were re-evaluated based on the inclusion criteria. A codebook for data extraction from the articles meeting the eligibility criteria was developed as previously described [84].

### Bioinformatic analysis

The miRNA-target gene relationships were collected from four resources, including miRTarbase, a database of experimentally validated miRNA-gene interactions [86], and three databases for predicted miRNA-gene interactions (miRanda [87], PITA [88] and TargetScan [89]). The Fisher’s exact test was used to test the significance level of the shared genes between miRNA target genes and mouse CL-associated genes. The Benjamini–Hochberg method was used for multiple test correction [69].

### Animals

C57BL6/J mice were obtained from The Jackson Laboratory. All mice were maintained in the animal facility of UTHealth. The protocol was reviewed and approved by the Animal Welfare Committee (AWC) and the Institutional Animal Care and Use Committee (IACUC) of UTHealth.

### Cell culture

Primary MELM cells were obtained from the maxillary process, a developing lip region, at E10.5 and E11.5, and cultured in Dulbecco’s Modified Eagle Medium (DMEM) supplemented with 10% fetal bovine serum (FBS), penicillin/streptomycin, L-glutamine, beta-mercaptoethanol, and non-essential amino acids. O9–1 cells were cultured under a conditioning medium obtained from STO cells (a mouse embryonic fibroblast cell line), as previously described [90]. Cells were plated on 96-well cell culture plates at a density of 5000/well and treated with mimic for negative control, miR-124-3p, let-7a-5p, let-7b-5p, let-7c-5p, and let-7d-5p (mirVana miRNA mimic, ThermoFisher Scientific), or with an inhibitor for negative control or miR-124-3p (mirVana miRNA mimic, ThermoFisher Scientific), using Lipofectamine RNAiMAX transfection reagent (ThermoFisher Scientific) and according to the manufacturer’s protocol (3 pmol mimic or inhibitor with 0.3 μl transfection reagent in 100 μl DMEM). Cell proliferation assays were performed using the cell counting kit 8 (Dojindo Molecular Technologies, Gaithersburg, MD).

### Immunoblotting

Immunoblots were performed as described previously [91], using a rabbit polyclonal antibody against cleaved caspase 3 (Cell Signaling Technology) and a mouse monoclonal antibody against GAPDH (MilliporeSigma).

### Quantitative RT-PCR

Total RNA isolated from either MELM cells (*n* = 6 per treatment group), the maxillary process, or the medial nasal process (n = 6 per developmental stage) was dissected with the QIAshredder and RNeasy mini or miRNeasy mini extraction kit (QIAGEN), as previously described [92]. The following PCR primers were used for further specific analysis: *Bmpr1a*, 5′-CCCCTGTTGTTATAGGTCCGT-3′ and 5′-TTCACCACGCCATTTACCCA-3′; *Cdc42*, 5′-ATGTGAAAGAAAAGTGGGTGCC-3′ and 5′-GATGCGTTCATAGCAGCACAC-3′; *Ift88*, 5′-TAGGATCAGGCGTCGCTTCT-3′ and 5′-GCAGTTACGGGAGGTCTTCT-3′; *Lrp6*, 5′-ATTATTGTCCCCGGATGGGC-3′ and 5′-ACTGCCTGCCGGTTTGTT-3′; *Pbx3*, 5′-CATCGGCGACATCCTCCAC-3′ and 5′-TGTGAATTCATTACATGCCTGTTCA-3′; *Tgfbr1*, 5′-GGCCGGGCCACAAACA-3′ and 5′-CTGAAAAAGGTCCTGTAGTTGGG-3′; *Zeb1*, 5′-GGAGGTGACTCGAGCATTTAGA-3′ and 5′-ACTCGTTGTCTTTCACGTTGTC-3′; and *Gapdh*, 5′-AACTTTGGCATTTGGAAGG-3′ and 5′-ACACATTGGGGGTAGGAACA-3′. Expression of miR124-3p (mmu480901) was measured using the Taqman Advanced miRNA Assays kit (ThermoFisher Scientific). Each expression level was normalized with miR-191-5p (ID 477952) expression.

### Statistical analysis

A two-tailed Student’s *t* test was applied for the statistical analysis. A *p* value < 0.05 was considered statistically significant. For all graphs, data were parametric and represented as mean ± standard deviation (SD).

## Supplementary information

**Additional file 1:** The information of the databases searched.

**Additional file 2:** Characterization of primary nasal cells isolated from E11.5 medial nasal process. (**A**) Cell proliferation assays in nasal cells treated with negative control (control, light blue), miR-124-3p (orange), let-7a-5p (gray), let-7b-5p (yellow), let-7c-5p (blue), and let-7d-5p (light green). ** *p* < 0.01, *** *p* < 0.001. (**B**, **C**) Expression of miR-124-3p (B) and its target genes (C) in the medial nasal process (NP) at E10.5 to E13.5. * *p* < 0.05, ** *p* < 0.01, *** *p* < 0.001.

## Data Availability

All data from this study are available as supplemental information.

## Abbreviations

Bmpr1a: bone morphogenetic protein receptor type 1A
CL: cleft lip
CL/P: cleft lip with/without cleft palate
CNC: cranial neural crest
DMEM: Dulbecco’s modified Eagle medium
FGF: fibroblast growth factor
MELM: mouse embryonic lip mesenchymal
miRNA: microRNA
TGFβ: transforming growth factor β
