# Supplementary material for: MicroRNA-124-3p suppresses mouse lip mesenchymal cell proliferation through the regulation of genes associated with cleft lip in the mouse
Source: BMC Genomics. 2019 Nov 14;20:852. doi: 10.1186/s12864-019-6238-4 (PMC6854646; doi:10.1186/s12864-019-6238-4)
Supplement: Supplementary file 1 — Additional file 1: The information of the databases searched. [file 12864_2019_6238_MOESM1_ESM.docx]

**Additional file 1.** The information of the databases searched.

**Table S1.** Summary of the databases searched

| **Table** | **Vendor/ interface** | **Database** | **Date searched** | **Database update** | **Searcher(s)** |
| --- | --- | --- | --- | --- | --- |
| 1a | Ovid | Medline^®^ | 5/12/2016 | In-Process & Other Non-Indexed Citations; Daily; 1946 to Present | Helena M. VonVille, Dima Summakia |
| 1b | National Library of Medicine | PubMed | 5/13/2016 | May 13, 2016 | Helena M. VonVille, Dima Summakia |
| 1c | Ovid | Embase | 5/16/2016 | 1980 to 2016 Week 19 | Helena M. VonVille, Dima Summakia |

**Table S1a:** Ovid Medline search strategy

**Provider/Interface** Ovid

**Database** Medline^®^

**Date searched** May 12, 2016

**Database update** In-Process & Other Non-Indexed Citations; Daily; 1946 to Present

**Search developer(s)** Helena M. VonVille, Dima Summakia

**English only?** Yes

**Date restrictions?** 1980-2016

| 1 | Cleft Lip |
| --- | --- |
| 2 | (harelip or harelips or cheiloschisis or ((cleft or clefting) adj3 lip)).ti,ab,kw. |
| 3 | lip/ and (clefting or cleft).ti,ab,kw. |
| 4 | 1 or 2 or 3 |
| 5 | ge.fs. |
| 6 | Chromosome Deletion/ or Chromosome Mapping/ or Crosses, Genetic/ or gene amplification/ or Gene Deletion/ or Gene Expression Regulation, Developmental/ or gene expression regulation/ or gene expression/ or genetic diseases, inborn/ or genetic diseases, x-linked/ or Genetic Linkage/ or Genetic Predisposition to Disease/ or genome-wide association study/ or homeodomain proteins/ or molecular epidemiology/ or mutagenesis/ or Mutation/ or Polymorphism, Single Nucleotide/ or protein modification, translational/ or protein processing, post-translational/ or RNA processing, post-transcriptional/ or Sequence Analysis, DNA/ or Signal Transduction/ or Transcription Factors/ or transcription, genetic |
| 7 | (chromosomal or chromosome or chromosomes or gene or genes or genetic or genetics or genome or genomes or mutation or mutations or protein or proteins).ti,ab,kw. |
| 8 | MicroRNAs |
| 9 | (microRNA or microRNAs or miRNA or miRNAs).ti,ab,kw. |
| 10 | 5 or 6 or 7 or 8 or 9 |
| 11 | 4 and 10 |
| 12 | mice/ or mice, inbred strains/ or Mice, Inbred C57BL/ or mice, mutant strains/ or mice, transgenic/ or mice, knockout |
| 13 | (mouse or mice).ti,ab,kw. |
| 14 | Disease Models, Animal |
| 15 | 12 or 13 or 14 |
| 16 | 11 and 15 |
| 17 | limit 16 to (English language and yr="1980 - 2016") |

**Table S1b:** PubMed search strategy

**Provider/Interface** National Library of Medicine

**Database** PubMed

**Date searched** May 13, 2016

**Database update** May 13, 2016

**Search developer(s)** Helena M. VonVille, Dima Summakia

**English only?** Yes

**Date restrictions?** 1980-2016

| 1 | Cleft Lip[mesh:noexp] |
| --- | --- |
| 2 | (harelip[tiab] OR harelips[tiab] OR cheiloschisis[tiab] OR ((cleft[tiab] OR clefting[tiab]) AND lip[tiab])) |
| 3 | lip[mesh:noexp] AND (clefting[tiab] OR cleft[tiab]) |
| 4 | #1 OR #2 OR #3 |
| 5 | ge[sh] |
| 6 | Chromosome Deletion[mesh:noexp] OR Chromosome Mapping[mesh:noexp] OR Crosses, Genetic[mesh:noexp] OR gene amplification[mesh:noexp] OR Gene Deletion[mesh:noexp] OR Gene Expression Regulation, Developmental[mesh:noexp] OR gene expression regulation[mesh:noexp] OR gene expression[mesh:noexp] OR genetic diseases, inborn[mesh:noexp] OR genetic diseases, x-linked[mesh:noexp] OR Genetic Linkage[mesh:noexp] OR Genetic Predisposition to Disease[mesh:noexp] OR genome-wide association study[mesh:noexp] OR homeodomain proteins[mesh:noexp] OR molecular epidemiology[mesh:noexp] OR mutagenesis[mesh:noexp] OR Mutation[mesh:noexp] OR Polymorphism, Single Nucleotide[mesh:noexp] OR protein modification, translational[mesh:noexp] OR protein processing, post-translational[mesh:noexp] OR RNA processing, post-transcriptional[mesh:noexp] OR Sequence Analysis, DNA[mesh:noexp] OR Signal Transduction[mesh:noexp] OR Transcription Factors[mesh:noexp] OR transcription, genetic[mesh:noexp] |
| 7 | (chromosomal[tiab] OR chromosome[tiab] OR chromosomes[tiab] OR gene[tiab] OR gene[tiab]s OR genetic[tiab] OR genetics[tiab] OR genome[tiab] OR genomes[tiab] OR mutation[tiab] OR mutations[tiab] OR protein[tiab] OR proteins[tiab]) |
| 8 | MicroRNAs[mesh:noexp] |
| 9 | (microRNA[tiab] OR microRNAs[tiab] OR miRNA[tiab] OR miRNAs[tiab]) |
| 10 | #5 OR #6 OR #7 OR #8 OR #9 |
| 11 | #4 AND #10 |
| 12 | mice[mesh:noexp] OR mice, inbred strains[mesh:noexp] OR Mice, Inbred C57BL[mesh:noexp] OR mice, mutant strains[mesh:noexp] OR mice, transgenic[mesh:noexp] OR mice, knockout[mesh:noexp] |
| 13 | (mouse[tiab] OR mice[tiab]) |
| 14 | Disease Models, Animal[mesh:noexp] |
| 15 | #12 OR #13 OR #14 |
| 16 | #11 AND #15 |
| 17 | #16 AND English[la] AND 1980:2016[dp] |

**Table S1c:** Ovid Embase search strategy

**Provider/Interface** Ovid

**Database** Embase

**Date searched** 5/16/2016

**Database update** 1980 to 2016 Week 19

**Search developer(s)** Helena M. VonVille, Dima Summakia

**English only?** Yes

**Date restriction** 1980-2016

| 1 | cleft lip |
| --- | --- |
| 2 | (harelip or harelips or cheiloschisis or (cleft adj3 lip)).ti,ab,kw. |
| 3 | congenital malformation |
| 4 | craniofacial malformation |
| 5 | (craniofacial abnormalities or craniofacial defects or craniofacial disorders or craniofacial malformations).ti,ab,kw. |
| 6 | 3 or 4 or 5 |
| 7 | lip |
| 8 | (lip or lips).ti,ab,kw. |
| 9 | 7 or 8 |
| 10 | 6 and 9 |
| 11 | 1 or 2 or 10 |
| 12 | mutant mouse strain/ or mouse mutant/ or mouse strain/ or akita mouse/ or "db/db mouse"/ or nude mouse/ or "ob/ob mouse"/ or quaking mouse/ or reeler mouse/ or scid beige mouse/ or scid mouse/ or shiverer mouse |
| 13 | mouse |
| 14 | transgenic mouse/ or genetically engineered mouse strain/ or 3xtg mouse/ or 5xfad mouse/ or big blue mouse/ or cystic fibrosis transmembrane conductance regulator mouse/ or gpt delta mouse/ or immortomouse/ or lcat mouse/ or mmp9 mouse/ or msod1 mouse/ or mutamouse/ or oncomouse/ or p-glycoprotein deficient mouse/ or pcsk9 mouse/ or sad mouse/ or tg2576 mouse |
| 15 | knockout mouse/ or low density lipoprotein receptor ko mouse/ or p-glycoprotein deficient mouse/ or "pxr-/-mouse"/ or "rag1-/- mouse" |
| 16 | (mouse or mice).ti,ab,kw. |
| 17 | disease model |
| 18 | 12 or 13 or 14 or 15 or 16 or 17 |
| 19 | 11 and 18 |
| 20 | chromosome deletion/ or chromosome deletion 13/ or chromosome deletion 22q11/ or chromosome deletion 4/ or chromosome deletion 5/ or chromosome deletion x/ or chromosome deletion y/ or interstitial chromosome deletion/ or philadelphia 1 chromosome |
| 21 | chromosome map |
| 22 | cross breeding/ or backcrossing/ or outcrossing |
| 23 | gene amplification/ or amplicon/ or ligase chain reaction/ or loop mediated isothermal amplification/ or multiplex ligation dependent probe amplification/ or multiplex polymerase chain reaction/ or nucleic acid amplification/ or nucleic acid sequence based amplification/ or reverse transcription loop mediated isothermal amplification/ or reverse transcription polymerase chain reaction/ or sequence characterized amplified region/ or telomeric repeat amplification protocol |
| 24 | gene mutation/ or allelic imbalance/ or deletion mutant/ or frameshift mutation/ or "gain of function mutation"/ or gene deletion/ or gene disruption/ or gene insertion/ or gene loss/ or indel mutation/ or "loss of function mutation"/ or missense mutation/ or mutator gene/ or nonsense mutation/ or null allele/ or point mutation/ or splicing defect |
| 25 | gene expression/ or antigen expression/ or "chromatin assembly and disassembly"/ or crispr cas system/ or enzyme induction/ or enzyme repression/ or epistasis/ or expression vector/ or gene expression regulation/ or gene overexpression/ or gene product/ or gene regulatory network/ or gene repression/ or gene silencing/ or genetic epigenesis/ or heterologous expression/ or nuclear reprogramming/ or position effect variegation/ or posttranscriptional gene silencing/ or protein induction/ or receptor down regulation/ or receptor upregulation/ or RNA interference/ or stable expression/ or transactivation/ or transient expression |
| 26 | genetic disorder/ or chromatid aberration/ or chromosome breakage/ or laminopathy |
| 27 | chromosome aberration/ or autosome aberration/ or chromosomal instability/ or mosaicism/ or nondisjunction/ or numerical chromosome aberration/ or sex chromosome aberration/ or structural chromosome aberration |
| 28 | genetic association |
| 29 | homeodomain protein/ or nucleic acid binding protein |
| 30 | molecular epidemiology |
| 31 | site directed mutagenesis/ or mutagenesis |
| 32 | mutation |
| 33 | DNA polymorphism/ or amplified fragment length polymorphism/ or restriction fragment length polymorphism/ or single nucleotide polymorphism |
| 34 | protein processing/ or protein modification |
| 35 | RNA processing/ or RNA cleavage/ or RNA editing |
| 36 | DNA sequence |
| 37 | signal transduction/ or intracellular signaling |
| 38 | transcription factor/ or genetic transcription |
| 39 | (chromosomal or chromosome or chromosomes or gene or genes or genetic or genetics or genetic or genetics or genome or genomes or mutation or mutations or protein or proteins).ti,ab,kw. |
| 40 | microRNA/ or small untranslated RNA/ or microRNA 1/ or microRNA 100/ or microRNA 101/ or microRNA 107/ or microRNA 10b/ or microRNA 122/ or microRNA 124/ or microRNA 125b/ or microRNA 126/ or microRNA 132/ or microRNA 141/ or microRNA 143/ or microRNA 145/ or microRNA 146a/ or microRNA 155/ or microRNA 15a/ or microRNA 15b/ or microRNA 16/ or microRNA 182/ or microRNA 192/ or microRNA 200/ or microRNA 200a/ or microRNA 200b/ or microRNA 200c/ or microRNA 205/ or microRNA 20a/ or microRNA 20b/ or microRNA 21/ or microRNA 210/ or microRNA 214/ or microRNA 22/ or microRNA 221/ or microRNA 222/ or microRNA 223/ or microRNA 24/ or microRNA 26a/ or microRNA 29/ or microRNA 29a/ or microRNA 29b/ or microRNA 31/ or microRNA 34a/ or microRNA 375/ or microRNA 9 |
| 41 | (microRNA or microRNAs or miRNA or miRNAs).ti,ab,kw. |
| 42 | 20 or 21 or 22 or 23 or 24 or 25 or 26 or 27 or 28 or 29 or 30 or 31 or 32 or 33 or 34 or 35 or 36 or 37 or 38 or 39 or 40 or 41 |
| 43 | 19 and 42 |
| 44 | limit 43 to (English language and yr="1980 - 2016") |
| 45 | 44 not conference.pt. |
| 46 | 45 not medline.cr. |
