# Supplementary material for: MicroRNA-124-3p suppresses mouse lip mesenchymal cell proliferation through the regulation of genes associated with cleft lip in the mouse
Source: BMC Genomics. 2019 Nov 14;20:852. doi: 10.1186/s12864-019-6238-4 (PMC6854646; doi:10.1186/s12864-019-6238-4)
Supplement: Supplementary file 2 — Additional file 2: Characterization of primary nasal cells isolated from E11.5 medial nasal process. (A) Cell proliferation assays in nasal cells treated with negative control (control, light blue), miR-124-3p (orange), let-7a-5p (gray), let-7b-5p (yellow), let-7c-5p (blue), and let-7d-5p (light green). ** p < 0.01, *** p < 0.001. (B, C) Expression of miR-124-3p (B) and its target genes (C) in the medial nasal process (NP) at E10.5 to E13.5. * p < 0.05, ** p < 0.01, *** p < 0.001. [file 12864_2019_6238_MOESM2_ESM.pdf]

Additional file 2. Suzuki et al.

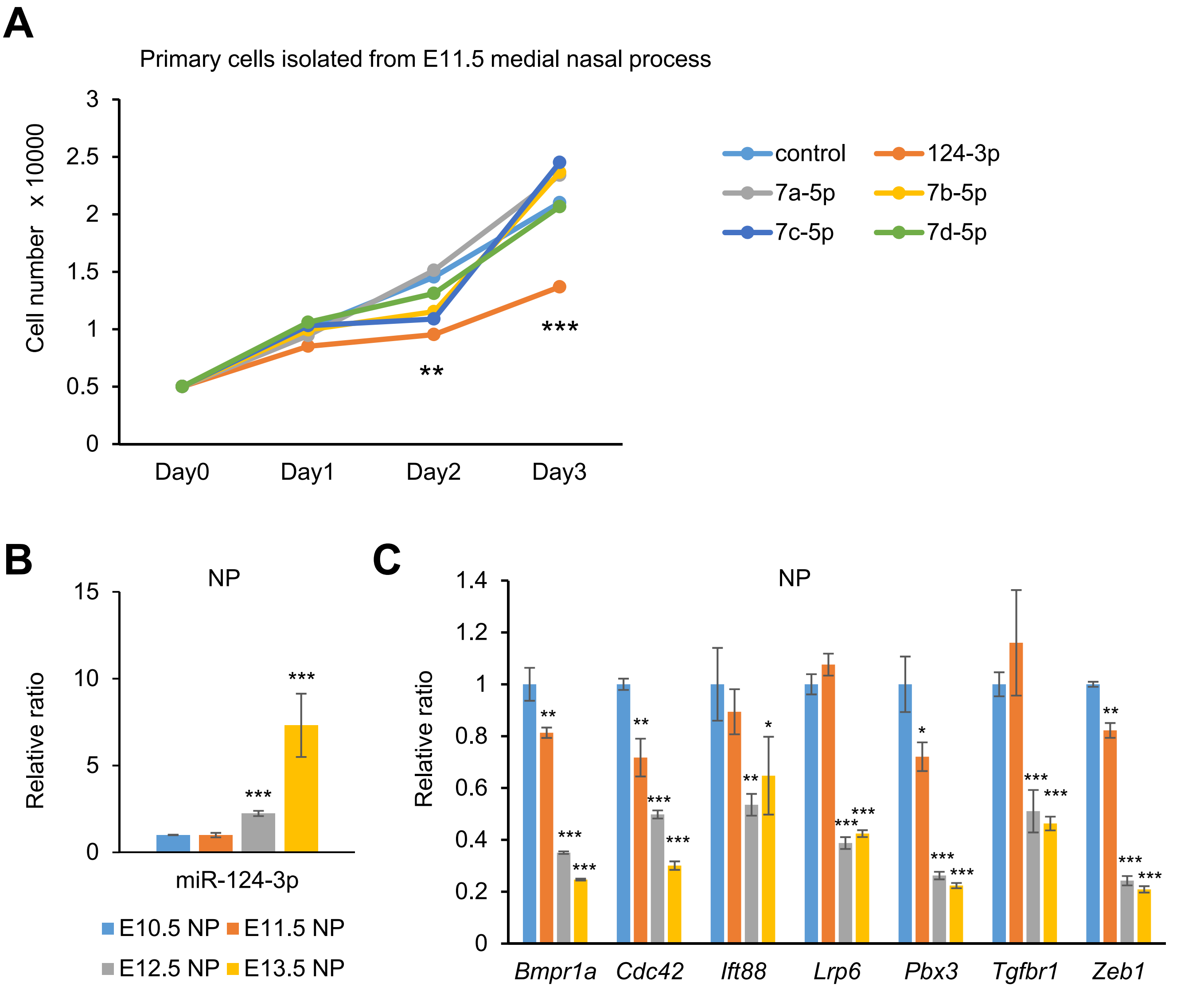

Additional file 2. Characterization of primary nasal cells isolated from E11.5 medial nasal process. (**A**) Cell proliferation assays in nasal cells treated with negative control (control, light blue), miR-124-3p (orange), let-7a-5p (gray), let-7b-5p (yellow), let-7c-5p (blue), and let-7d-5p (light green). ** $p < 0.01$, *** $p < 0.001$. (**B, C**) Expression of miR-124-3p (B) and its target genes (C) in the medial nasal process (NP) at E10.5 to E13.5. * $p < 0.05$, ** $p < 0.01$, *** $p < 0.001$.
